# Editorial: Bone inside-out and outside-in signals: Control of body homeostasis

**DOI:** 10.3389/fendo.2022.1120215

**Published:** 2023-01-06

**Authors:** Uma Sankar, Lucas R. Brun, Lilian I. Plotkin

**Affiliations:** ^1^ Department of Anatomy, Cell Biology & Physiology, Indiana University School of Medicine, Indianapolis, IN, United States; ^2^ Indiana Center for Musculoskeletal Health, Indiana University School of Medicine, Indianapolis, IN, United States; ^3^ Bone Biology Laboratory, School of Medicine, Rosario National University, Rosario, Argentina; ^4^ Consejo Nacional de Investigaciones Científicas y Técnicas (CONICET), Rosario, Argentina; ^5^ Roudebush Veterans Administration Medical Center, Indianapolis, IN, United States

**Keywords:** bone, osteocyte, osteoclast, osteoblast, endocrine signals

## Summary

Bone mass is controlled by the coordinated actions of osteoblasts and osteoclasts, cells responsible for bone formation or bone resorption, respectively. It is now clear that the actions of these two cell types, as well as their differentiation from the corresponding precursor cells, is modulated by osteocytes, the cells embedded in the bone matrix. Recent findings indicate that in addition to their role in controlling bone mass, bone cells act as endocrine cells, and all cells produce factors able to regulate the function of distal organs, including muscle, pancreas, kidney and brain, among others. Conversely, hormones and growth factors produced by cells, other tissues modulate bone modeling and remodeling by altering osteoblast, osteoclast and osteocyte functions. Further, more recently studies provided evidence for a role of the gut microbiome on bone homeostasis. Understanding the molecular signature of bone cells, and how these cells are affected by circulating factors has provided new means to treat conditions with altered bone mass and strength.

The purpose of this Research Topic Issue was to discuss different aspects of bone interactions with other tissues, both inside-out (from bone cells to other organs) and outside-in (from other cells/tissues to bone cells). This Research Topic included 20 articles from which 7 are originals and 13 reviews. The following is a summary of the articles included in the Research Topic Issue.

## Role of osteocytes as endocrine cells

Osteocytes, previously believed to be inert cells embedded in the bone matrix, are emerging as key regulators of bone homeostasis, as well as cells producing molecules that signal to other tissues and organs. Fibroblast growth factor 23, of osteocytic fibroblast growth factor 23 (FGF23) is produced and secreted primarily by osteocytes both as an active molecule and as truncated, inactive fragments that can be detected in the circulation. The authors describe the response of osteocytes to increased phosphate and 1,25(OH)2 vitamin D, leading to the production of FGF23. In turn, FGF23 activate renal receptors to control circulating phosphate levels. The synthesis and secretion of FGF23 is regulated by positive and negative signals. Osteocytes can “sense” phosphate levels by a mechanism not completely understood, but that appears to involve the type III sodium phosphate co-transporter PiT2 (Slc20a2). In addition to high phosphate levels, FGF23 is positively regulated by pro-inflammatory molecules and iron deficiency, hypoxia and erythropoietin, TGFβ, calcineurin and NFAT, as well as transcriptional regulation by parathyroid hormone, and calcitriol. FGF23 can be also negatively regulated by PHEX, DMP1, insulin and IGF-1. In addition to these regulations, the level and activity of FGF23 is regulated by intracellular proteolytic enzymes. Upon enzymatic cleavage, FGF23 becomes inactive. Another important emerging function of osteocytes is the control of bone matrix properties. Thus, osteocytes have the ability to communicate and modulate the generation and function of osteoblasts and osteoclasts, which can in turn alter the properties of bone matrix through changes in bone formation and resorption. In addition, it has been proposed that osteocytes themselves have the ability to remodel the perilacunar space, changing the composition of the bone matrix. Studies *in vitro* described in this article used osteocytic cell lines, and their response to endocrine, paracrine, and mechanical stimuli has been studied using 2D and 3D systems. *In vivo* studies focused mainly on the perilacunar remodeling during lactation and hibernation, with emphasis in the consequences of these conditions on mineral composition and structure, as well as in the role of osteocytic genes on collagen degradation. More recent studies have shown the role of osteocytes in osteolytic metastasis. While reviewing the current understanding on how on how osteocytes regulate the growth of bone metastatic tumors bone metastatic tumors, the authors proposed both protective and enhancing effect of osteocytes on tumor growth and metastasis. For example, osteocytes can promote prostate cancer cell proliferation, migration, and invasion *via* the release of growth-derived factor 15 (GDF15), and osteocyte apoptosis within lytic multiple myeloma lesions leads to increased sclerostin and RANKL production, generating a negative bone remodeling balance. This is followed by activation of Notch signaling and increased myeloma cell proliferation. Other studies have shown an inhibitory effect of osteocytic Cx43 on cancer cell growth, mediated at least in part but the release of ATP though Cx43 hemichannels.

## Osteocalcin and its endocrine functions

 Osteoblasts and osteocytes are proposed to be endocrine cells, producing and releasing osteocalcin and sclerostin, which in turn are able to act on cells and tissues outside the bone, fulfilling the definition of “hormones”. Osteocalcin (γ-carboxyglutamic acid, Gla, protein) is synthesized as pro-hormone by osteoblasts, it is later cleaved γ-carboxylated, which increases the affinity of the protein for the mineral component of the bone matrix. γ-carboxylated osteocalcin can be stored within the bone and later be released by bone resorbing osteoclasts. Full deletion of the osteocalcin gene has shown disparate effects on bone mass and strength. These differences could be due to different manners for gene deletion, differences in the mouse genetic background, sex, age in which the analyses were made. Discrepancies were also reported on the role of osteocalcin in energy metabolism, with disparate consequences on glucose levels, body weight, muscle weight, and male reproduction depending on the osteocalcin deficiency model used. Studies were also performed in mice lacking the Bglap (exon 4) and Bglap2 genes to determine the role of osteocalcin in the central nervous system, prompted by observations during animal handling. Further studies showed that mice deficient in osteocalcin exhibit hippocampal atrophy and dramatic changes in neurotransmitter levels in the central nervous system. These effects are ascribed to the lack of circulating maternal uncarboxylated osteocalcin, which can cross the placenta and fetal blood-brain barrier. In another set of studies using the same mouse model, it was shown that osteocalcin regulates output of parasympathetic neurons under acute stress response, and that sympathetic input in this condition increases osteocalcin production by osteoblasts. These effects of osteocalcin have not been tested in the other osteocalcin deficiency models.

Osteocalcin secreted by osteoblasts and released from the bone matrix by osteoclasts has been shown to regulate muscle mass independently of its effects on systemic energy homeostasis. Circulating osteocalcin levels in older postmenopausal women with osteoporosis correlate with skeletal muscle mass and their OAK-score-related risk of falling. This study also found plasma carboxylated osteocalcin levels to positively correlate with lean body mass and muscle function. Thus, circulating osteocalcin could mediate bone-muscle crosstalk, and its levels could be predictive of muscle function and fall risk in older post-menopausal women. Further, bone-secreted factors such as lipocalin influence hypothalamic feeding behavior, suggesting the existence of a bone-brain axis. Factors responsible for neuropsychological symptoms were evaluated in the serum of patients with primary hyperparathyroidism. The study found that serum osteocalcin levels positively correlate with parathyroid hormone (PTH) levels and negatively correlate with State-Trait Anxiety Inventory (STAI). Thus, serum osteocalcin was associated with psychological performance in patients with primary hyperparathyroidism and could mediate bone-brain crosstalk.

## Outside-in signaling: hematopoietic and endocrine cells regulate bone cell function

Wnt signaling plays a critical role in skeletal homeostasis. The skeletal role for Wnt secreted by hematopoietic cells was examined by conditionally deleting Wnt modifying enzyme PORCN using *Vav-Cre* transgenic mice. Hematopoietic *Porcn* deletion did not affect normal skeletal development but delayed fracture healing. These mice possessed fewer osteoclasts at the fracture callus causing a delay in callus remodeling. Calcitonin is a small peptide hormone secreted by the thyroid gland in response to increases in serum calcium levels. It inhibits osteoclastic bone resorption and associated release of skeletal calcium. Calcitonin also enhances osteoblastic bone formation, but the underlying mechanism was not fully understood, especially because the calcitonin receptor is present only in osteoclasts and not osteoblasts. It is now reported that calcitonin induces bone formation by increasing the expression of the clastokine Wnt10b in osteoclasts in ovariectomized rat models of osteoporosis, indicating a thyroid-bone crosstalk.

## Lipid regulation by bone-produced molecules

Sclerostin is a potent inhibitor of Wnt signaling that is mainly produced by mature osteocytes. Through its interaction with LRP4/5/6, sclerostin has been shown to stimulate adipogenesis by inhibiting Wnt signaling. Although no evidence of changes in adipose tissue in patients with absence of sclerostin has been reported, clinical studies showed positive correlations between serum sclerostin levels and fat mass and the incidence of metabolic disorders. Studies *in vitro* and in animal models support the role of sclerostin on adipogenesis and fat mass. Another set of studies showed that sclerostin has direct effects in the kidney, regulating calcium excretion and the synthesis of 1α,25(OH)_2_D, thereby contributing to the control of mineral homeostasis. Others have shown that sclerostin can affect the cardiovascular system *via* inhibition of vascular calcification, and that its inhibition can lead to elevated risk of cardiovascular adverse events. Serum sclerostin was found to be significantly lower in patients with non-alcoholic fatty liver disease (NFALD) patients compared to non-NFALD patients. In NFALD patients, serum sclerostin levels negatively correlated with metabolic parameters including fatty liver index and triglyceride levels. These findings indicate the existence of a bone-liver crosstalk playing a role in abnormal bone metabolism in NFALD patients and suggest the utility of serum sclerostin as a biomarker for bone disease in NFALD patients.

In addition of utilizing glucose as source of energy, osteoblasts are able to internalize and metabolize lipids. In a review by Alekos et al. the mechanism by which disruption of lipid metabolism in osteoblasts affects the skeleton and whole body lipid homeostasis is discussed. In particular, the evidence supporting the requirement for fatty acid oxidation in osteoblasts and the detrimental consequences of its inhibition on the skeleton and bone repair following fractures. Further, fatty acid oxidation is regulated by bone anabolic treatments, and the review describes how Wnt and parathyroid hormone signaling. In particular, LRP5 deletion and expression of a gain of function mutant lead to opposite effects on lipids markers, with increase and decrease in fat mass and serum triglycerides and free fatty acid, respectively. These effects of Wnt signaling are dependent on β-catenin expression, suggesting that canonical Wnt signaling is involved in the regulation of lipid metabolism by osteoblasts. Evidence also suggests that parathyroid hormone and 1,25 dihydrocycholecalciferol influence fatty acid oxidation at least *in vitro*/ex vivo, and that intermittent PTH administration might induce the release of fatty acids from adipocyte to fuel osteoblast anabolic activity. The review further discusses the consequences of dyslipidemia in the skeleton, and how hyperlipidemia impacts the response of bone to anabolic signals. Thus, increase lipids induce the release of Wnt inhibitors, and accumulation of oxidized lipids inhibits PTH signaling. In addition, dyslipidemia leads to insulin resistance in osteoblasts, which might contribute to bone loss associated with this condition. PPARγ, a transcription factor that regulates adipogenesis has been identified as a potential link between hyperlipidemia and bone loss. Metabolic diseases detrimentally affect the bone. Both hyperglycemia and ketogenic diet diminished bone mass, microstructure and strength due to enhanced osteoclast and diminished osteoblast activity.

## Bone-muscle crosstalk in the masticatory system

Further evidence of the role of bone as a endocrine organ, and the crosstalk with skeletal muscle is described in the masticatory system. In addition of biomechanical interaction between bone and muscle, the authors describe the role of myostatin, IGF-1 and IL-6 as mediators of the communication between the two tissues. The authors further discuss the clinical evidence supporting the biochemical interaction between muscle and bone at the masticatory system. For example, muscle-released IL-6 is proposed to mediate mandibular remodeling in tempomandibular disorders and potentially, the deleterious effects on mandibular bone following muscle paralysis by botulinum toxin. Conversely, bone-derived osteocalcin has been linked to masticatory muscle hypertrophy. Thus, osteocalcin can regulate IL-6 levels in skeletal muscle, which in turn upon release is able to modify bone remodeling, creating a feedback loop between bone and muscle. Other biochemical mediators of the bone-muscle interaction are sclerostin and RANKL, produce and released by osteocytes in bone, and with catabolic potential in skeletal muscle cells, can be further evaluated as complementary therapeutical targets for highly prevalent oral diseases such as periodontitis.

## Osteoimmunology

Osteoimmunology is emerging as a new interdisciplinary field to explore the shared molecules and interactions between the skeletal and immune systems. Here, a review summarizes the regulatory roles of T lymphocytes in osteoporosis and the development of T cell therapy for osteoporosis from osteoimmunology perspective. The inflammatory response is related to bone healing due to chronic inflammation can lead to impaired fracture healing. This review summarizes the principles of inflammation and provide an update on cellular interactions and immunomodulation for optimal bone healing. Interleukin-6 (IL-6) is a pro-inflammatory mediator that plays a key role in obesity-induced loss of bone microarchitecture by inducing senescence of mesenchymal stem cells. Bone regeneration is promoted by human amniotic mesenchymal stem cells due to paracrine functions on immune-regulation, anti-inflammation and vascularized tissue regeneration. The review focus on the therapeutic effects and mechanisms of this cells in promoting bone regeneration in joint diseases and bone defects. Inflammasomes are multiprotein complex of the innate immune system responsible for secretion of pro-inflammatory cytokines. The chronic inflammatory microenvironment induced by aging or estrogen deficiency activates the NLRP3 inflammasome. The review about NLRP3 Inflammasome highlights the function of NLRP3 inflammasome in osteoporosis as a role in the pathogenesis of osteoporosis by affecting the differentiation of osteoblasts and osteoclasts. providing information on new strategies for managing osteoporosis. Further, the TNF-mediated inflammatory osteoclastogenesis is also considered providing potential therapeutic strategies to selectively treat inflammatory bone resorption, without undesirable effects on normal bone remodeling or immune response in disease settings. HIV infection leads toward an inflammatory state associated with chronic and immune dysregulation activation and involve the OPG/RANKL/RANK system. It also provides antiretroviral therapy-related detrimental effects on bone metabolism.

## Bone-nervous system interactions

Orthopaedic pain management remains a clinical and societal challenge. The skeleton is well-innervated, but its function remains under-appreciated. Understanding the signaling between bone and nerve during skeletal development, fracture healing and aging will lead to a better comprehension of skeletal pain and better therapeutic strategies to alleviate orthopaedic pain.

## Author contributions

All authors listed have made a substantial, direct, and intellectual contribution to the work and approved it for publication.

